# Fc-Engineering for Modulated Effector Functions—Improving Antibodies for Cancer Treatment

**DOI:** 10.3390/antib9040064

**Published:** 2020-11-17

**Authors:** Rena Liu, Robert J. Oldham, Emma Teal, Stephen A. Beers, Mark S. Cragg

**Affiliations:** 1 GlaxoSmithKline Research and Development, Stevenage SG1 2NY, UK; rena.x.liu@gsk.com; 2 Antibody and Vaccine Group, Centre for Cancer Immunology, Cancer Sciences, Faculty of Medicine, University of Southampton, Southampton SO171BJ, UK; robert.oldham@soton.ac.uk (R.J.O.); E.L.Teal@soton.ac.uk (E.T.); msc@soton.ac.uk (M.S.C.); 3 Cancer Research UK Centre, Cancer Sciences, Faculty of Medicine, University of Southampton, Southampton SO171BJ, UK

**Keywords:** Fc-engineering, Fc gamma receptor, antibody immunotherapy, complement, antibody-dependent cellular cytotoxicity (ADCC), antibody-dependent cellular phagocytosis (ADCP)

## Abstract

The majority of monoclonal antibody (mAb) therapeutics possess the ability to engage innate immune effectors through interactions mediated by their fragment crystallizable (Fc) domain. By delivering Fc-Fc gamma receptor (FcγR) and Fc-C1q interactions, mAb are able to link exquisite specificity to powerful cellular and complement-mediated effector functions. Fc interactions can also facilitate enhanced target clustering to evoke potent receptor signaling. These observations have driven decades-long research to delineate the properties within the Fc that elicit these various activities, identifying key amino acid residues and elucidating the important role of glycosylation. They have also fostered a growing interest in Fc-engineering whereby this knowledge is exploited to modulate Fc effector function to suit specific mechanisms of action and therapeutic purposes. In this review, we document the insight that has been generated through the study of the Fc domain; revealing the underpinning structure-function relationships and how the Fc has been engineered to produce an increasing number of antibodies that are appearing in the clinic with augmented abilities to treat cancer.

## 1. Introduction

Antibodies are a central component of immunity, serving to prevent and control pathogen spread whilst providing long-lasting protection from re-infection. Classically described as Y shaped molecules, antibodies contain two identical fragment antigen binding (F(ab)) arms coupled through a hinge to a fragment crystallizable (Fc) domain. The F(ab) arms provide specificity and mediate target antigen binding, whilst the Fc facilitates engagement with immune effector functions (i.e., cellular immunity through interaction with Fc receptors and the complement system through binding to C1q) [1,2,3]. The Fc also prolongs half-life in circulation through its interaction with FcRn [4]. Furthermore, although the F(ab) domains can deliver therapeutic activity, for example by preventing a pathogen:host binding event leading to infection, the majority of antibody effector function, particularly for IgG1, is delivered by the Fc.

This knowledge has led to a concerted effort in the antibody field to understand which parts of the Fc regulate engagement with the various arms of the immune effector system and subsequently whether modifications can deliver enhanced activity. This review summarizes the advances in these endeavors and provides an overview of where they have been or soon will be, translated into clinical investigation for the treatment of cancer.

Central to the advancement of antibodies as clinical therapeutics was the development of monoclonal antibodies (mAb), following the advent of hybridoma technology [5]. This breakthrough permitted the production of antibodies with a single specificity, facilitating approvals through the regulatory bodies for use in humans, as well as enabling structure:function studies of homogeneous antibody preparations and analysis of post-translational modifications such as glycosylation [6]. This was followed by the explosion in molecular biology techniques that have since enabled the manipulation of antibody molecules to a previously unheralded extent. Together, these advances, paved the way for the subsequent era of Fc engineering. Within this, antibodies targeting cancer have been at the forefront.

As discussed above, the principle property of the Fc domain is to evoke immune effector function through interaction with Fc receptors and the complement initiator, C1q. Although further antibody classes are now entering the clinic (see other articles in this collection), almost all therapeutic antibodies approved to date are of the IgG class and so much of our current understanding of antibody effector function has evolved from their study. Below (Section 1.1, Section 1.2 and Section 1.3), we summarize the three critical interactions that the IgG Fc mediates in modulating antibody effector function and behavior.

### 1.1. FcγR Binding Leading to Cellular Effector Functions

IgG molecules bind to Fc gamma receptors (FcγR). Humans possess 6 different FcγR: hFcγRI, hFcγRIIA, hFcγRIIB, hFcγRIIC, hFcγRIIIA and hFcγRIIIB [1,7], which bind IgG at the cell surface but with varying affinity. hFcγRI contains three extracellular immunoglobulin domains and is the only high-affinity FcγR, able to bind monomeric IgG, whilst the other FcγR contain two extracellular immunoglobulin domains and are low to intermediate affinity, only binding IgG in immune complex or when bound to a pathogen or cell surface.

Broadly, FcγR are functionally characterized into two main types; activatory and inhibitory. Activatory FcγR, (hFcγRI, hFcγRIIA, hFcγRIIC, hFcγRIIIA), contain or are associated with an immune tyrosine activation motif (ITAM). In two of the human activatory receptors (hFcγRIIA and hFcγRIIC) the ITAM is contained within the intracytoplasmic domain of the FcγR itself whilst on the remaining activatory receptors it is contained within an associated FcR γ chain. In mice, which contain 4 FcγR, all 3 activatory FcγR require the FcR γ chain for both expression and signaling [1,8]. FcγRIIB by contrast contains an immune tyrosine inhibitory motif (ITIM) and is the only inhibitory FcγR in both humans and mice. In addition to these activatory and inhibitory FcγR, there is one GPI-linked hFcγR, which lacks both ITAM and ITIM motifs, hFcγRIIIB. Its function is less clear, with reports variously supporting roles as a neutral or decoy receptor, modulating FcγRIIA-mediated neutrophil degranulation and ROS production [9]. FcγRI is a high affinity receptor for IgG and as such is largely considered to be saturated by IgG in the circulation [10]. Despite evidence suggesting that it is capable of mediating anti-tumor immunotherapy in certain B16 mouse models, it is still considered a minor player in immunotherapy [11]. As such there has been comparatively little effort to engineer antibodies with altered binding to FcγRI. Notwithstanding hFcγRIIIB, the general concept is that where both activatory and inhibitory FcγR exist on a given cell type (common on myeloid cells), then the relative binding of IgG to each expressed FcγR is integrated to modulate the cellular response. In practice the activatory to inhibitory or A:I FcγR binding ratio of Fc and/or FcγR cellular expression profiles are a good estimate of subsequent cellular activity [12,13], with high A:I ratios providing robust responses. One of the key determinants is antibody isotype, with each isotype providing a diverse FcγR binding profile and cellular response. For example, hIgG1 binds strongly to most FcγR and hIgG4 much less so. The FcγR family and their isotype interactions have been extensively described elsewhere [14] and so only specific points are highlighted within this review.

Genetic variations of hFcγR can also affect expression and function [15,16]. In particular, certain single-nucleotide polymorphisms (SNPs) affect binding to IgG. Notably, a valine (V) to phenylalanine (F) SNP at position 158 effects binding of FcγRIIIA to IgG1, IgG2 and to a lesser extent IgG3, whereby V158 has an increased affinity for hIgG1 compared to F158, resulting in increased activation [4,17]. Similarly, a histidine (H) to arginine (R) SNP in FcγRIIA at position 131 affects binding to hIgG2 (and to a lesser extent to hIgG1), with increased binding seen for H131 compared to R131.

FcγRs clearly have the potential to impact the efficacy of anti-cancer antibodies, especially through activation of Fc-effector functions such as antibody-dependent cellular cytotoxicity (ADCC) and antibody dependent cellular phagocytosis (ADCP). ADCC is thought to be principally mediated by NK cells [18]. NK cells bind to the arrayed Fc of antibody-labelled tumor cells, predominantly through a single activatory FcγR (FcγRIIIA). Binding of IgG to FcγRIIIA can activate NK cells and stimulate the release of lytic granules containing molecules such as perforin and granzymes, ultimately leading to lysis of the target cell [19]. Many clinically-relevant anti-cancer antibodies such as rituximab, herceptin and cetuximab have been shown to evoke NK-mediated ADCC in vitro.

ADCP is mediated by phagocytic cells, such as macrophages, monocytes and neutrophils and is triggered when antibodies interact with the corresponding activatory FcγRs [20]. The anti-CD30 antibody, SGN-30, used for the treatment of several hematological diseases, such as Hodgkin disease, is an example of an anti-cancer antibody that can stimulate ADCP in vitro [21]. Rituximab and other anti-CD20 antibodies are also known to elicit potent ADCP both in vitro and in vivo in various animal models [22,23]. In these systems, removal of macrophages removes all target deleting capacity of these antibodies, highlighting the importance of this Fc-mediated effector mechanism.

### 1.2. C1q Engagement and Induction of Complement Mediated Effector Functions

Another important interaction mediated by the Fc is its binding to the first component of complement, C1q. Once sufficient Fc molecules engage the 6 globular heads of C1q, it initiates a proteolytic cascade of complement proteins in serum leading to release of anaphylatoxins such as C3a and C5a and the formation of the membrane attack complex at the target cell surface, capable of complement dependent cytotoxicity (CDC) [24]. Initially, the evidence for the importance of CDC in anti-cancer mAb effector functions was demonstrated through in vitro studies where it was found that tumor cells overexpress complement defence molecules such as CD55 and CD59; and blocking these could enhance the tumor killing capacity of rituximab [25]. Further evidence suggesting CDC plays a part in anti-cancer antibody efficacy includes increased consumption of complement components when chronic lymphocytic leukemia (CLL) cells are treated with rituximab; and a reduced capacity of complement-depleted CLL patient sera to lyse targets cells [26]. Rituximab was also shown to be ineffective in C1q-deficient mice in xenograft experiments with human lymphoma cells, suggesting complement activation is required for tumor deleting activity in vivo [27]. However, xenograft models may fail to predict complement activity in patients as the target cells may be hyper-sensitive to xeno-complement [28,29]. Accordingly, experiments with syngeneic models such as hCD20 transgenic mice demonstrated that anti-CD20 antibodies engineered without complement-activating function had equivalent B cell depletion capacity as wild-type (WT) anti-CD20 antibodies [30]. This indicates that complement activity is not required, at least in mice. In agreement, genetic deletion of C1q or C3, does not appreciably impair deletion of hCD20 transgenic B cells with various anti-CD20 mAb [31]. Clinical data demonstrating that expression of complement defence molecules on tumor cells could not predict clinical outcome for follicular lymphoma (FL) patients after rituximab treatment, further suggests CDC is not a key determinant of successful tumor clearance after rituximab [32].

### 1.3. FcRn Interaction Leading to Long Half-Life

The third major interaction mediated by the Fc is with the bramble receptor or neonatal FcR (FcRn) reviewed in Reference [4]. This receptor differs substantially from the FcγR family both in terms of structure and function. Structurally, the receptor more closely resembles that of the major histocompatibility complex (MHC) and it does not contain or associate with domains capable of signal transduction [4,33]. It was originally identified as important for the transportation of IgG across the placenta from mother to fetus, however its broader role in maintaining IgG homeostasis was soon established [34,35]. Unlike the direct effector functions outlined above, this interaction is responsible for providing the long half-life of antibodies, which for IgG is over 20 days in humans and 9 days in mice [36,37]. Serum proteins, including IgG are continually endocytosed by monocytes and endothelial cells. FcRn is able to scavenge internalized IgG and return it to the circulation. During this process, FcRn binds the CH2-CH3 interface of the Fc in a pH dependent manner. The conserved histidine residues (H310 and H435) in the CH2-CH3 domains of IgG become protonated at the acidic pH (<pH6.5) present within intracellular organelles such as lysosomes, which allows tight binding between FcRn and IgG and resulting protection [38,39]. Once the FcRn:IgG complex recycles to the extracellular environment the neutral pH allows the IgG to be re-released into the bloodstream; accounting for its long serum half-life. The importance of this process is highlighted in FcRn deficient mice, where mIgG half-life is reduced >6-fold, from 9 to 1.4 days, an effect that is overcome by the introduction of a human FcRn transgene [36].

All of the properties and activities discussed above are delivered through a highly evolved and fine-tuned series of molecular interactions between the Fc and the molecules detailed, interfacing antibodies with the powerful effector functions of the immune system and enabling their long half-life. It is therefore unsurprising that numerous attempts have been made to augment or modify each of these interactions. Summaries of the antibody variants that have been developed to modify these interactions are provided in the ensuing Tables and Figures. A more detailed review of the variants, their discovery and the major implications are expanded in the following sections according to their functional output (effector cell function, complement-mediated, FcγRIIB binding and half-life extending) and are discussed below. In addition, we consider those mAb where reduced effector function has been engineered to improve therapeutic responses.

## 2. Fc Engineering to Enhance Antibody Effector Cell Function

As detailed above, FcγRs are central to IgG effector functions. Therefore, Fc engineering strategies have been employed to increase affinity for FcγRs, in order to enhance Fc-effector functions such as ADCC and ADCP and thereby increase the efficacy of anti-cancer antibodies. These strategies have been summarized in Table 1 and are discussed below.

**Figure 1:**
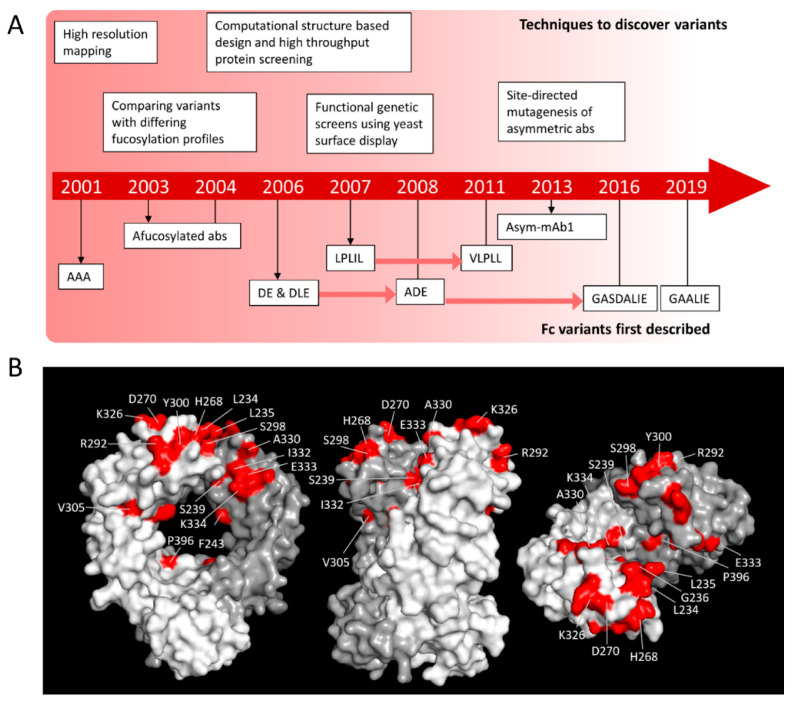
Fragment crystallizable (Fc) modifications which augment antibody effector function. (**A**) Timeline of events detailing techniques used to discover variants with enhanced effector cell function and when the Fc variants were first described [40,41,42,43,44,45,46,47,48]. Letters refer to the common notation of the introduced amino acid substitutions as discussed in the main text. (**B**) Previously identified point mutations within the Fc that impact effector cell function annotated on a hIgG1 Fc crystal structure model (PDB: 3AVE; [49,50]) and detailed in Table 1.

Over the last 2–3 decades, techniques have been employed to determine the amino acids involved in the binding of FcγRs to IgG. In a seminal study in 2001, Shields et al. performed high resolution mapping using alanine scanning mutagenesis [40] to determine the residues within hIgG1 required for binding hFcγR and hFcRn (Figure 1A). The binding site for hFcγRs was found in the lower hinge and proximal CH2 regions of hIgG1 [40]. The key residues involved in these interactions have subsequently been the focus for mutational studies and are highlighted in Figure 1B. These studies paved the way for a greater understanding of how IgG molecules interact with FcγRs.

### 2.1. Amino Acid Substitutions to Enhance Effector Cell Function

Guided by the above studies, numerous groups subsequently introduced amino acid substitutions into the Fc region of antibodies to increase the A:I binding ratio and therefore augment their hFcγR-mediated effector functions (summarized in Figure 1A). Based upon their initial observations, Shields et al. designed variants incorporating amino acid substitutions that displayed enhanced binding to FcγRIIIA but reduced binding to FcγRIIB [40]. This included the variant S298A/E333A/K334A which showed enhanced ADCC compared to WT IgG when introduced into the humanized IgG1 anti-IgE mAb E27 [40].

Groups also adopted computational structure-based modelling coupled with high throughput protein screening in order to identify amino acid substitutions to enhance hFcγR-mediated effector functions. For example, the anti-CD52 hIgG1, alemtuzumab, with S239D/I332E or S239D/A330L/I332E mutations was found to have increased binding affinity to both allotypes of FcγRIIIA (although greater for the V158 allele) and FcγRIIB [42,51]. Consequently, ADCP was demonstrated to be modestly improved whilst ADCC was significantly enhanced by introducing these amino acids into the anti-HER2 hIgG1 antibody, trastuzumab [42,51]. Importantly, although both S239D/I332E and S239D/A330L/I332E mutations can successfully enhance ADCC, only S239D/I332E variants retain CDC capacity whereas this is reduced for S239D/A330L/I332E variants when compared to WT hIgG1 [42,51]. The potential implication of this difference is discussed below. In vivo, rituximab incorporating the S239D/I332E substitutions results in a significantly lower IC50 for B cell depletion in cynomolgus monkeys (going from 10 μg^−1^kg^−1^ for WT rituximab to 0.2 μg^−^1 kg^−1^ for the S239D/I332E variant) [42].

Using similar screening techniques, other variants were discovered that had greater affinity for FcγRIIA relative to FcγRIIB to enhance ADCP [43]. This included the G236A mutation which provided a 6-7-fold enhancement in binding to both allotypes of FcγRIIA, whilst not significantly altering affinity for FcγRIIB or FcγRIIIA when compared to WT hIgG1 [43]. Notably, the IIA/IIB ratios (Kd (FcγRIIA) / Kd (FcγRIIB)) were 15 and 12 (for H131 and R131 allotypes, respectively) for the G236A variant, compared to 2.7 and 2.5 for WT hIgG1 [43]. As a result, this single amino acid mutation was introduced into an anti-EpCAM hIgG1 antibody, resulting in a modest increase in ADCP whilst retaining ADCC levels comparable to the WT antibody [43]. However, the G236A mutation was found to reduce FcγRI affinity by around 7-fold which could make this variant suboptimal therapeutically [43]. Therefore, other mutations were incorporated to rescue binding to FcγRI including the S239 mutation to generate the variant, G236A/S239D/I332E [43]. G236A/S239D/I332E hIgG1 demonstrated substantially enhanced binding to all FcγRs with binding enhanced ~30-fold for the FcγRIIA-R131 allotype, albeit with only minor increases in ADCC and ADCP observed (Table 1) [43].

More recently, antibodies carrying the G236A/A330L/I332E and G236A/S239D/A330L/I332E mutations were generated and assessed for their ability to enhance FcγR-mediated effector functions [44,45]. Both were found to have enhanced binding to activatory FcγRs (FcγRIIA and FcγRIIIA), with G236A/A330L/I332E hIgG1 also displaying lower binding for the inhibitory FcγRIIB, whereas G236A/S239D/A330L/I332E hIgG1 had enhanced binding to FcγRIIB [44,45]. In vitro, G236A/A330L/I332E hIgG1 had superior ADCC capability compared to the WT [44]. When incorporated into an anti-CD20 hIgG1, both variants demonstrated significantly higher depletion of hCD20^+^ B cells in hCD20/hFcγR transgenic mice when compared to WT anti-CD20 hIgG1 [44,52]. However, not only did G236A/S239D/A330L/I332E hIgG1 show a reduced protein thermal shift measurement when compared to the WT (~55 °C vs ~68 °C, respectively) but it also possessed considerably reduced half-life in the hFcγR transgenic mice which is hypothesized to be because of its increased affinity for FcγRs and decreased in vivo stability [44]. This finding highlights two important considerations when Fc-engineering; first there may be a limit for usefully increasing binding to low-affinity hFcγRs and secondly the unanticipated effects on other Fc properties.

To identify novel human IgG1 Fc regions with increased binding to FcγRIIIA but reduced binding to FcγRIIB in order to enhance the A:I ratio and enhance Fc effector functions, functional genetic screens using yeast surface display techniques have also been employed (Figure 1A) [46]. The combination of mutations, F243L/R292P/Y300L/V305I/P396L was discovered this way and demonstrated 10-fold enhanced binding to FcγRIIIA compared to WT rituximab with minimal enhancement in binding to FcγRIIA and FcγRIIB [41].

### 2.2. Glycoengineering to Enhance Effector Cell Function

Glycoengineering has also been shown to be a powerful tool for modifying Fc-mediated cellular effector functions. This follows realization that the glycans attached at the N297 residue of the Fc are critical for efficient binding to FcγR and C1q [53,54,55,56,57]. Although initially unclear why these carbohydrates are so critical, it was recently shown that the hIgG1 Fc N297 glycan is involved in intramolecular interactions between carbohydrate and amino acid that stabilize the Fc C′ strand and C′E loop and orientate the hFcγRIIIA interface for Fc binding [58]. With regards effector cell function, glycosylation at the N297 site has been found to be critical for the induction of ADCC due to its ability to stabilize the Fc region and keep it in an ‘open’ conformation [59,60]. Different combinations of oligosaccharides can often occur at this site and many IgGs often incorporate N-acetylglucosamine (GlcNAc), which, in combination with other residues, forms a core carbohydrate [59,60]. A residue that is often part of the core GlcNAc is fucose [60]. Due to steric hindrance, this fucose impairs optimal interaction of IgG with FcγRIIIA [53,59]. Therefore, eliminating fucose at this site has been widely investigated to enhance binding to FcγRIIIA and augment effector cell function.

In 1999, it was found that overexpressing the N-acetylglucosaminyltransferase III (GnTIII) enzyme in CHO cells could produce hIgG1 antibodies with significantly reduced fucosylation [61]. Engineering a chimeric anti-neuroblastoma IgG1 (chCE7) in this way led to higher ADCC [61]. This mechanism of generating antibodies with reduced fucosylation was marketed as GlycoMab^®^ technology, developed by the Swiss Biotech, Glycart, and later acquired by Roche [62].

Another method for generating afucosylated antibodies stemmed from a study in 2003, showing that humanized antibodies generated in a rat hybridoma YB2/0 cell line had 50-fold higher ADCC activity compared to those produced in CHO cell lines (Figure 1A) [63]. Monosaccharide composition and oligosaccharide profiling analysis revealed that antibodies produced in YB2/0 cell lines had a lower fucose content when compared to those produced in CHO cell lines [63]. This prompted interest in generating antibodies with even lower fucose content. Most notably, a technique now licensed as POTELLIGENT^®^ from Kyowa Hakko Kirin Co., uses FUT8 knockout CHO cells to generate afucosylated antibodies [64]. Initial studies looking at afucosylated antibodies generated this way exhibited a 13-fold increase in binding to FcγRIIIA-F158 and a 5-fold increase in ADCC (Table 1) [41]. An afucosylated CCR4 antibody generated in this way was found to be significantly more potent in human PBMC-engrafted SCID mouse models as well as in suppressing tumor growth in syngeneic and xenograft mouse models compared to its WT counterpart [65].

Since then there have been many other efforts to glycoengineer antibodies to enhance effector cell function, reviewed at length previously [66,67]. However, afucosylation remains the most prevalent glycoengineering technique for enhancing effector cell function and antibodies incorporating these technologies are being assessed in clinical trials, with some now approved (Table 2). It is however important to note that preparations of glycoengineered antibodies can have a high degree of heterogeneity, a key hurdle for gaining regulatory approval.

### 2.3. Anti-Cancer Antibodies with Enhanced Effector Cell Function Entering the Clinic

Numerous antibodies engineered to exhibit augmented Fc effector functions are currently being assessed in clinical trials with a summary of those approved and in late stage clinical trials shown in Table 2. Due to the majority of clinically approved anti-cancer antibodies exerting their effects through the direct deletion of a target cell population, enhancing their effector function has been the area of mAb engineering that has seen the most clinical progress. The first such antibody approved for use in cancer was obinutuzumab in 2015, an anti-CD20 antibody developed using Glycart GlycoMAb^®^ technology to elicit low fucosylation. In key head-to-head studies in CLL and FL, obinutuzumab in combination with chemotherapy improved progression free survival (PFS) compared to rituximab plus chemotherapy [69,70]. However, in a study of 1418 diffuse large B cell lymphoma (DLBCL) patients, obinutuzumab in combination with CHOP chemotherapy offered no advantage over R-CHOP, suggesting that the increased FcγRIII-binding as a result of glycoengineering may only provide benefit in certain diseases [71,72]. It is also important to note that in these trials the dosing levels and regimen differed between the rituximab and obinutuzumab arms. An additional caveat to these comparisons is that rituximab and obinutuzumab have differences in their mechanism of action. Rituximab is a type I anti-CD20 mAb and can redistribute CD20 into lipid rafts to induce potent CDC, whilst obinutuzumab is a type II mAb, which lacks these properties but can evoke a non-apoptotic form of direct cell death [31,73]. Similarly, rituximab is rapidly internalized from the cell surface whilst obinutuzumab is not, again, owing to their respective type I/II natures [74,75]. Thus, it is difficult to determine how much of the improvement in efficacy can be attributed to Fc glycoengineering.

Ublituximab is another anti-CD20 antibody engineered to have low fucose content [76]. In the GENUINE phase 3 study of high risk CLL patients, ublituximab became the first anti-CD20 mAb to provide additional patient benefit to the BTK inhibitor ibrutinib, with a significant improvement in overall response rate (ORR) and PFS [76]. In a comparable trial, rituximab in combination with ibrutinib failed to improve PFS compared to ibrutinib alone suggesting that the enhanced effector function of ublituximab may provide patient benefit [77]. Additionally, the UNITY-CLL trial compared ublituximab plus the PI3K inhibitor umbralisib to obinutuzumab plus chlorambucil. The trial was stopped early due to superior efficacy in the ublituximab group [78]. However, with both antibodies administered in entirely different combinations, the relative contributions of the different antibodies (and more specifically their Fc modifications) to therapeutic outcome cannot be directly assessed. Another anti-CD20 antibody, ocaratuzumab containing the P247I/A339Q mutations to enhance ADCC has also been investigated in the clinic [79]. However, despite a phase 2 trial (NCT00003874) completing in 2011, results have not been published.

Moving away from anti-CD20 mAb, the anti-CCR4 mAb, mogamulizumab, is Food and Drug Administration (FDA) approved for use in adult T-cell leukemia/lymphoma (ATLL) as well as mycosis fungoids (MF) and Sézary syndrome, 2 subtypes of cutaneous T cell lymphoma (CTCL) [80]. In the largest trial ever conducted in CTCL, mogamulizumab gave a superior PFS compared to the HDAC inhibitor vorinostat (7.7 v 3.1 months) [81]. In ATLL, mogamulizumab also had a favorable ORR compared to an investigators choice control arm but did not improve PFS [82]. This is in contrast to an earlier but smaller study in ATLL which yielded an improvement in PFS, although this may reflect the importance of ATLL subtype in response to mogamulizumab [83].

Tafasitamab (MOR208), is a CD19 targeting antibody approved by the FDA in 2020 for use in relapsed/refractory DLBCL. It was engineered with the S239D/I332E mutation to enhance ADCC via an increase in affinity for FcγRIIIA. In the RE-MIND study, DLBCL patients receiving tafasitamab in combination with lenalidomide were compared to matched historical data for patients receiving lenalidomide alone. The trial concluded that the combination arm had a superior ORR (67.1%) than lenalidomide monotherapy (34.2%) [84]. Following these promising results, tafasitamab is currently undergoing direct comparison to rituximab in combination with bendamustine in the B-MIND trial [85]. Top line results for this study are expected at the beginning of 2022. Despite targeting a different antigen to rituximab, results may give an insight into the improvement in clinical outcome that can be gained by enhancing FcγRIIIA binding.

As already indicated, direct comparisons between WT and Fc-modified hIgG1 reagents are lacking, making definitive evidence for the importance of Fc-engineering difficult to obtain. Indeed, in addition to the positive indications detailed above, not all antibodies engineered for enhanced effector function have given the expected patient benefit. For example, ocrelizumab, an anti-CD20 mAb which shares an overlapping CD20 epitope with rituximab, has been engineered for enhanced ADCC activity [86]. In a phase I/II trial of FL patients previously treated with rituximab, it achieved an ORR of 38% [87]. However, the same ORR was achieved in a similar study of patients retreated with rituximab [88]. As a result, development of ocrelizumab for hematological malignancies was suspended. Despite this, the antibody has subsequently been approved for use in multiple sclerosis. Similarly, CetuGEX, an afucosylated version of the anti-EGFR mAb cetuximab failed to improve PFS compared to the native antibody. Moreover, its use was associated with an increase in infusion related events [89]. This study suggests that, at least for certain targets, the potential benefit of enhancing effector cell activation may need to be balanced against increased toxicity.

Despite these complications, Fc-enhanced mAb have seen success in solid tumors. Macrogenics recently reported interim results from their anti-HER2 antibody margetuximab, engineered for enhanced FcγRIIIA binding through 5 amino acid substitutions (L235V/F243L/R292P/Y300L/P396L) [47]. In a direct comparison, margetuximab plus chemotherapy resulted in a modest improvement in PFS compared to trastuzumab plus chemotherapy (5.8 v 4.9 months) [90]. Perhaps most importantly, the improved response was most pronounced for patients carrying at least 1 copy of the low affinity 158F FcγRIIIA allele (6.9 v 5.1 months). Patients expressing this allelic form of FcγRIIIA have typically had a worse response to a number of direct targeting antibodies including trastuzumab [91]. Engineering mAb for enhanced effector cell function may therefore allow patients in these poor response groups to reap greater benefit from mAb therapy. Following these promising results, margetuximab is being evaluated in combination with checkpoint inhibitor blockade through anti-PD1 mAb for gastric and gastroesophageal junction cancer, with the trial estimated to complete in 2024 [92].

Antibodies targeting checkpoint inhibitors such as CTLA-4 were originally thought to induce therapeutic responses solely through blockade of the receptor on T cells. However, subsequent research suggested that deletion of Treg may also contribute to their activity, although the extent of this contribution is disputed [93,94]. The emergence of Treg deletion as a potential mechanism of anti-CTLA4 mAb activity has provided the impetus to develop anti-CTLA4 mAb with enhanced target cell deletion. To date, two anti-CTLA4 antibodies engineered for enhanced Fc-function have entered the clinic; zalifrelimab (AGEN1884) and AGEN1181. In vitro analysis suggested zalifrelimab could improve Treg cell deletion [95]. However interim analysis of a phase I trial suggested that when combined with anti-PD1 mAb, responses were comparable to that seen with the unmodified anti-CTLA-4 mAb ipilimumab, albeit with a favorable safety profile [96].

## 3. Fc Engineering to Enhance Antibody-Mediated Complement Dependent Cytotoxicity

**Figure 2:**
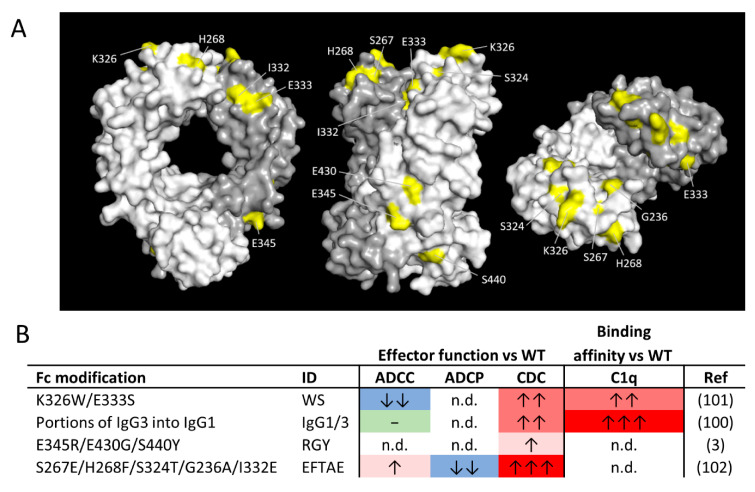
Fc modifications which increase complement-dependent cytotoxicity. (**A**) Previously identified point mutations within the Fc that modulate complement dependent cytotoxicity are annotated on a human IgG1 Fc crystal structure model (PDB: 3AVE; [49,50]). (**B**) Fold change in effector function or binding affinity to C1q in complement dependent cytotoxicity (CDC)-enhancing variants compared to wild type (WT) antibodies. Magnitude of effector function or binding affinity of variants were compared to WT antibodies of the same IgG isotype and F(ab) region. Each row depicts an independent experiment and therefore often independent methods, utilizing a different IgG backbone from a particular study as referenced in the last column. NB = no detectable binding, n.d. = no data, − = no change, ↑ <2 fold upregulation, ↑↑ 2–9.99 fold upregulation, ↑↑↑ 10–99.99 fold upregulation, ↓↓ 2–9.99 fold downregulation,.

Although the importance of C1q binding and CDC for the therapeutic activity of WT hIgG1 mAb is unclear, recent studies have indicated that antibodies engineered not to bind to FcγRs and bind C1q more strongly than WT IgG are capable of engaging powerful CDC and providing tumor control in pre-clinical models [97]. Similarly, seminal studies from Diebolder and Ugurlar indicate that powerful CDC requires efficient hexamerisation of hIgG1 at the cell surface, which can be augmented through appropriate amino acid mutations within the Fc [3,98]. Importantly, efficient CDC is constrained by specific target molecule requirements. For example, the target epitope has to be positioned relatively close to the cell membrane for efficient lysis with the density of the target antigen also important [99]. As a result of these very specific target requirements, whilst CDC may not be a critical component of WT antibody efficacy against cancer cells, it may be possible to deliver enhanced anti-tumor efficacy through this route. There are several CDC-enhancing Fc variants that have previously been described, summarized in Figure 2 [3,100,101,102]. As they have been the subject of excellent recent reviews [103,104,105], they will not be discussed further here.

## 4. Fc Engineering to Enhance Antibody Binding to FcγRIIB

**Figure 3:**
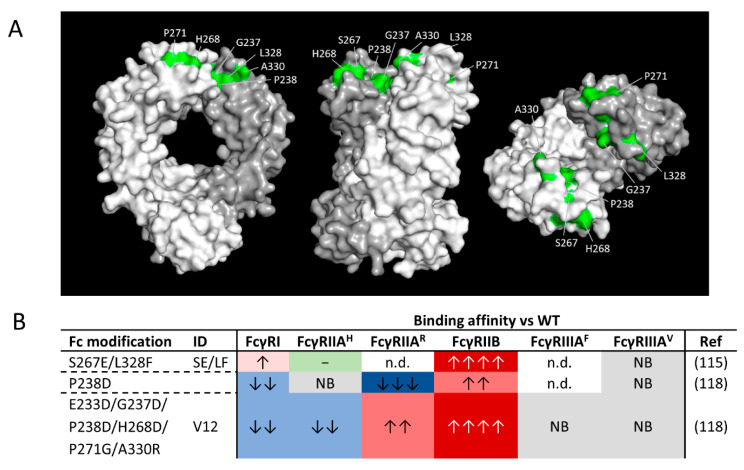
Fc modifications which enhance binding to FcγRIIB. (**A**) Previously identified point mutations within the Fc that impact binding to FcγRIIB annotated on a hIgG1 Fc crystal structure model (PDB: 3AVE; [49,50]). (**B**) Magnitude of binding affinity of variants were compared to WT antibodies of the same IgG isotype and F(ab) region. Each row depicts an independent experiment and therefore often independent methods, utilizing a different IgG backbone from a particular study as referenced in the last column. NB = no detectable binding, n.d. = no data, − = no change, ↑ < 2 fold upregulation, ↑↑ 2–9.99 fold upregulation, ↑↑↑↑ > 100 fold upregulation, ↓↓ 2–9.99 fold downregulation, ↓↓↓ 10–99.99 fold downregulation, **FcγRIIA^H^** FcγRIIA-H131, **FcγRIIA^R^** FcγRIIA-R131, **FcγRIIIA^F^** FcγRIIIA-F158, **FcγRIIIA^V^** FcγRIIIA-V158.

FcγRIIB is the only inhibitory FcγR expressed by humans and mice. For direct targeting, tumor-deleting antibodies, expression of FcγRIIB has been associated with reduced efficacy. For example, internalization of rituximab from the tumor cell surface has been associated with expression of FcγRIIB in vitro and high FcγRIIB has been associated with reduced activity of rituximab in vivo, presumably due to its ability to reduce ADCP [75,106]. Seminal studies by Clynes and Ravetch also demonstrated that mouse FcγRIIB impaired the activity of the breast cancer antibody trastuzumab [106,107]. However, in contrast, FcγRIIB has been shown to positively regulate the activity of immunomodulatory agonistic antibodies, through more effective clustering of mAb target receptors [108]. This was initially reported for agonistic anti-DR5 antibodies that require cross-linking by FcγRIIB in order to successfully elicit tumor control but has now been shown for multiple tumor necrosis factor receptor super-family (TNFRSF) members such as CD40, 4-1BB and OX40, at least in pre-clinical models [109,110,111,112,113,114]. Therefore, engineering antibodies to enhance binding to FcγRIIB has been of particular interest for agonistic anti-cancer antibodies. Data from these studies are summarized in Figure 3 and are described in detail in Section 4.1.

### 4.1. Amino Acid Substitutions Designed to Enhance FcγRIIB Binding

Combining computational structure-based modelling and high-throughput protein screening, Chu et al. screened over 900 variants to uncover antibody Fc domains with optimal FcγR binding profiles for immunostimulation, that is, displaying a low A:I ratio [115]. Through this screening strategy the group uncovered the amino acid substitutions S267E/L328F that when introduced into hIgG1, delivered a 430-fold increase in binding affinity to FcγRIIB with minimal changes in binding to FcγRI and FcγRIIA-H131 and eliminating binding to FcγRIIIA-V158 compared to WT hIgG1 [115]. S267E/L328F mutations were reported to facilitate the binding of engineered anti-OX40 antibodies to FcγRIIB and enhance agonism by over 2-fold in vitro [116]. In vivo, an S267E/L328F modified anti-CD40 hIgG2 antibody had enhanced ability to activate T cells in hFcγR/hCD40 transgenic mice when compared to either WT hIgG1 or hIgG2 variants [117].

In a variation of this approach, Mimoto et al. screened over 500 hIgG1 variants, replacing around 30 residues in the lower hinge and CH2 regions with other amino acids [118]. Through this process they discovered the mutation P238D that enhanced binding to FcγRIIB, while either completely abolishing or severely reducing binding to activatory FcγRs (FcγRI, FcγRIIA-H131, FcγRIIIA-V131) compared to WT hIgG1 [118]. The group then added further amino acid changes to P238 in order to enhance binding to FcγRIIB, including the mutations: G237D/H268D/P271G/A330R (termed V11) that enhanced binding to FcγRIIB by 40-fold when compared to the WT IgG1 [118]. However, it was a combination of 6 amino acid substitutions E233D/G237D/P238D/H268D/P271G/A330R (termed V12) that had the highest enhancement of binding to FcγRIIB at around 217-fold compared to the WT IgG1 [118]. V12 also has no detectable binding to either allotype of FcγRIIIA; reduction in binding to FcγRI and FcγRIIA-H131 and only slight enhancement of binding to FcγRIIA-R131 when compared to WT hIgG1 [118]. These V12 mutations were introduced into anti-OX40 antibodies and demonstrated >2-fold increased agonism in vitro as measured by an NF-κB reporter assay [48]. Since the discovery of mutations to enhance binding to FcγRIIB, there have been multiple anti-cancer antibodies that have incorporated these and are being assessed in clinical trials as discussed in Section 4.2.

### 4.2. Anti-Cancer Antibodies with Enhanced FcγRIIB Binding Entering the Clinic

As discussed above, there is growing evidence that immunostimulatory antibodies, particularly those targeting TNFRSF members can be further cross-linked by FcγRIIB, to augment receptor clustering and deliver effective agonism [110,112]. Certainly, this has been shown for anti-CD40 mAb, although it is important to recognize that this varies according to the specific mAb and the epitope on CD40 targeted [119]. Accordingly, LVGN7408, developed by Lyvgen Biopharma, has been engineered for increased FcγRIIB binding, although details of the exact modifications made have not been disclosed. Currently data is limited but its progress will be keenly monitored, particularly as increased agonism mediated through FcγRIIB has often been associated with elevated toxicity and pre-clinical data indicates that intra-tumoral administration was required for a different anti-CD40 mAb when it was optimized for FcγRIIB engagement through the V11 (G237D/P238D/H268D/P271G/A330R) mutations [120].

4-1BB (CD137) is another TNFRSF member, which is an attractive therapeutic target. Like, CD40 it is more effectively cross-linked and agonized by mAb that engage FcγRIIB [121] but like CTLA-4 its high relative expression on Treg makes it more complex to target therapeutically. For example, interaction of anti-4-1BB mAb with activatory FcγR leads to Treg deletion whilst cross-linking by FcγRIIB is required for efficient co-stimulation of CD8^+^ effectors, with both mechanisms giving anti-tumor activity in pre-clinical models [121]. Indeed, optimal anti-tumor activity was observed in mice with a mIgG2a antibody containing a hIgG2 hinge, providing efficient deletion whilst delivering optimal cross linking [121]. The anti-41BB mAb LVGN6051 is currently under investigation in combination with pembrolizumab. This antibody has been optimized for FcγRIIB interactions and was found to induce efficient T cell activation without the commonly observed liver toxicity [122]. However, activatory FcγR engagement has been abrogated, preventing Treg deletion as a mechanism of action. The efficacy of this antibody with a single mode of action remains to be seen and may provide additional useful mechanism of action data for subsequent anti-41BB mAb. An interesting alternative approach to overcome the requirement for FcγRIIB engagement for 4-1BB clustering has been through the development of bispecifics. In one format developed by Roche, one F(ab) arm targets 4-1BB whilst the other targets antigens that are highly expressed in the microenvironment of a specific tumor, such as CD19 or fibroblast activation protein (FAP) [123]. This has been reported to facilitate efficient receptor clustering without the need for FcγRIIB engagement.

## 5. Fc Engineering to Produce Antibodies without Fc Functions

**Figure 4:**
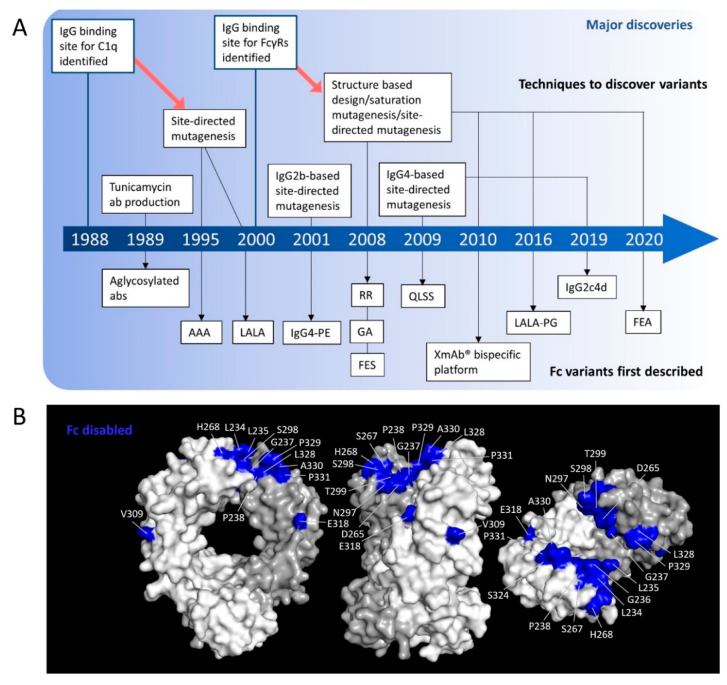
Fc modifications which reduce antibody effector function and/or FcγR binding. (**A**) Timeline of events detailing major discoveries, techniques used to discover variants that have a silenced Fc region and when the Fc variants were first described in the literature [57,115,124,127,128,129,130,132,133,134,135,136]. Letters refer to the common notation of the introduced amino acid substitutions as discussed in the main text. (**B**) Previously identified point mutations within the Fc that silence Fc functions annotated on a hIgG1 Fc crystal structure model (PDB: 3AVE; [49,50]) and detailed in Table 3.

In the preceding text, a number of approaches designed to augment Fc effector function have been described. However, engaging immune effectors through FcγR and complement interactions may be detrimental to certain mAb mechanisms of action and so Fc-null or -silenced antibodies may be desired. For example, with antibodies whose purpose is to block cell surface receptors or cytokines, Fc-effector functions are not required and may even be detrimental [105]. For anti-cancer antibodies, this is especially important in immune checkpoint inhibitor antibodies where target cell depletion is not desired and many clinically-approved anti-cancer checkpoint inhibitors have been selected with naturally low Fc effector function isotypes or are being designed with Fc-null or Fc-silenced formats as discussed later in this review. Fc-silencing techniques are summarized in Table 3 with key residues highlighted in Figure 4B.

### 5.1. Amino Acid Substitutions to Reduce Effector Function

After the characterization of the FcγR and C1q binding site within hIgG [137], various groups used site-directed mutagenesis in order to generate variants with reduced effector functions (Figure 4). In 1995, a L235A/G237A/E318A hIgG1 antibody was shown to be unable to bind to human cell lines expressing FcγRs resulting in an almost 4-fold reduction in ADCC when compared to WT hIgG [127]. In 2000, L234A/L235A Fc-disabling mutations were discovered in a similar manner by using site-directed mutagenesis of the C1q binding site [128]. OKT3 antibodies with hIgG1 and hIgG4 L234A/L235A Fc domains showed no detectable binding to the low affinity FcγRs and C1q and substantially diminished binding to FcγRI leading to a significant reduction in ADCC and CDC in comparison to WT hIgG1 or IgG4 [128].

Mutations were subsequently introduced at specific residues in hIgG1 known to interact with both FcγRs and C1q (see Figure 4B) [129]. These studies identified the amino acid substitutions L234F/L235E/P331S that led to reduced binding to the low affinity FcγRs (FcγRIIB and FcγRIIIA-F158 were not assessed) and no detectable binding to FcγRI [129]. In the same year, a combination of computational structure-based protein design and high-throughput protein screening of over 900 variants led to the discovery of the G236R/L328R mutation pair [115]. These mutations led to a reduction in or complete abrogation of, binding to the FcγRs measured (FcγRIIA-R131 and FcγRIIIA-F158 allotypes were not assessed) [115]. This study also uncovered the S267E substitution that reduced binding for all low affinity hFcγRs (NB: the FcγRIIA allele studied was H131; the FcγRIIIA allele studied was V158) [115]. In a subsequent study, a S267K substitution was combined with a series of mutations that were found in the lower hinge of hIgG2 (E233P/L234V/L235A mutations and a deletion of residue G236) and incorporated into a hIgG1 background [133,138]. The resulting antibody showed a lack of binding to all hFcγR investigated (hFcγRIIIA-F158 was not assessed) and these mutations became the backbone for which Xencor’s XmAb^®^ bispecific platform antibodies are generated [133]. Also, by analyzing the hFc-hFcγR interface, Schlothauer et al. identified a novel mutation, P329G, that was able to disrupt the interaction between hIgG and hFcγR. Coupled to the L234A/L235A mutations, thereby creating the triple mutant L234A/L235A/P329G, they produced a variant with no detectable binding to C1q or FcγRs, resulting in abrogated ADCC when introduced into a hIgG1 anti-EGFR antibody [130].

Many of these studies introduced amino acid substitutions at similar positions, therefore in 2020, Engelberts et al. combined several point mutations (N297Q, L234F, L235E, D265A, P331S) previously discovered to ablate Fc function [135] and assessed them in an anti-CD3-hIgG1. They found that the combination of L234F/L235E/D265A was the most potent at silencing the Fc region as demonstrated by no detectable binding to FcyRI, reduced binding to the low affinity FcyRs and reduced binding to C1q [135].

In an alternative approach, using crystal structures of the IgG1 Fc:FcγRIII complex, Sazinsky et al. found extensive contacts were made with the CH2 domain of IgG1, particularly in the Fc C′/E loop [124]. The group therefore created site saturation mutagenesis libraries centered about the Fc C′/E loop, theoretically encoding all amino acid combinations at residues 296–299, 297–299 and 297–300 in hIgG1. A series of mutants were subsequently generated and screened for reduced FcγR binding by flow cytometry [124], revealing that the S298G/T299A mutations abolished or significantly reduced binding to C1q and most FcγRs except for FcγRIIA-R131 and FcγRIIB [124].

### 5.2. Glycoengineering to Reduce Effector Function

Glycoengineering techniques have also been used to generate antibodies with reduced effector functions. For example, in 1989, antibodies were produced in the presence of tunicamycin to generate aglycosylated antibodies [57]. These aglycosylated antibodies were shown to have significantly less binding to FcγRI (~35-fold lower inhibition of RBC rosetting than wild type IgG1) and FcγRII [57]. Subsequently, aglycosylated variants targeted through specific amino acid substitutions were generated to produce Fc silenced antibodies (See Figure 4B and Table 3). As previously mentioned, the N297 glycan is central to the binding between hIgG1 and FcγRs and C1q, with structural data demonstrating how interactions between the hIgG1 Fc N297-glycan and FcγRIIIA are maintained [139]. Furthermore, it was also proposed that interactions between different hFcγRs, C1q and other hIgG isotypes are mediated in the same way [58]. Due to the importance of the N297 glycan for FcγR and C1q binding, amino acid mutations at this site, including N297A, N297Q and N297G, which remove this glycan, have been shown to reduce binding to all FcγRs and C1q, resulting in reduction of ADCC and CDC [124,125,126]. With regards the extent to which any of these Fc modifications are silenced, it should be noted that many of the aforementioned studies used flow cytometry and/or surface plasmon resonance (SPR) to assess FcγR binding. These methods typically rely on the use of soluble or monomeric versions of these antibodies, however, it was shown in 2009 that more physiologically relevant immune complexes should be considered to determine FcγR binding for hIgG isotypes [140], with immune-complexes displaying far higher avidity-mediated binding than monomeric formats. Lux et al. then extended this SPR assessment to evaluate various Fc-silenced antibody formats by generating complexes of antigen:Fc-silenced antibodies (in this case anti-TNP:TNP complexes) and observing binding to hFcγRs stably expressed on CHO cells, human B cell lines or peripheral blood mononuclear cells [141]. They found that aglycosylated hIgG1/2/3 antibodies, presented as large immune complexes retain WT levels of binding to FcγRIIA-131R [141], when previous studies using monomeric, aglycosylated hIgG1/3 showed no detectable binding [57,124]. Furthermore, they showed that hIgG1-N297A or enzymatically deglycosylated hIgG1 maintain binding to the high-affinity FcγRI, independent of immune-complex size [141], which is again contrary to previous reports assessing monomeric Fc-engineered antibodies [57]. This highlights the caution that must be taken when interpreting data from monomeric antibody:FcγR binding studies using Fc-silencing techniques and perhaps antibodies that incorporate Fc-engineering strategies as a whole.

### 5.3. hIgG4 and hIgG4 Fc Engineering to Reduce Effector Function

The simplest exponent of reduced Fc effector function is perhaps the use of hIgG4 as the choice of isotype. Although not Fc-engineering *per se*, it has long been known that different hIgG isotypes convey varying levels of effector function [142] which was later attributed to their differential binding to FcγRs and C1q [140,143,144]. In particular, the hIgG4 isotype is known to have low affinity for all FcγR, except FcγRI and has historically been favored for the development of Fc-inert antibodies [144]. In fact, hIgG4 antibodies are the second most approved class of antibody products behind hIgG1. For anti-cancer antibodies, the hIgG4 class is often used for checkpoint inhibitor antibodies such as those targeting PD-1. This is due to the relative inability of hIgG4 antibodies to stimulate ADCC or CDC, a desirable attribute for antibodies that target important adaptive immune cells required to elicit anti-tumor immunity [142].

However, despite these reduced Fc effector functions, hIgG4 antibodies are subject to the phenomenon of F(ab) arm exchange and so require specific amino acid substitutions to prevent this. F(ab) arm exchange involves formation of half antibody molecules comprising of one heavy and one light chain which can recombine with other half-molecules (for example in the serum) to form monovalent bispecific antibodies of unknown specificity [144]. It was found that a serine at position 228 plays a pivotal role in this phenomenon, by making inter-heavy chain disulphide bonds more susceptible to reduction, stimulating breakage of the covalent link between half-molecules and thus being a prerequisite for F(ab) arm exchange [145]. The importance of the serine at 228 was confirmed when a S228P substitution was shown to provide homogeneous hIgG4 when analyzed by SDS-PAGE. This substitution is commonly introduced in therapeutic hIgG4 antibodies to prevent F(ab) arm exchange [144,146].

Based upon its inherent lack of effector function, the human γ4 constant region has been the subject of various Fc-silencing approaches. In 2001, a group reconfigured a chimeric (primate/human) IgG1 anti-CD4 antibody, kiliximab, by exchanging the human γ1 region with that of human γ4, which resulted in reduced effector functionality [136]. To decrease residual FcγR binding further, they compared sequences of hIgG isotypes to the murine IgG2b isotype, which also has low FcγR binding activity, which uncovered an amino acid that differs from the hIgG4 sequence at position 235 [136]. Incorporating the mouse IgG2b residue (glutamic acid) into the hIgG4 antibody at this position, further minimized Fc effector function [136]. The resulting antibody with the S228P/L235E mutations was found to have either no or substantially reduced, binding to all FcγRs and C1q, resulting in no measurable ADCC [130]. In chimpanzees, the S228P/L235E anti-CD4 antibody (clenoliximab) failed to deplete CD4 T cells in contrast to WT hIgG4 [136].

Rather than replacing the whole constant region of hIgG1 with hIgG4 as above, various groups subsequently used site-directed mutagenesis to introduce specific amino acids from human γ4 into antibodies of other IgG isotypes. For example, An et al. found a combination of amino acid mutations—H268Q/V309L/A330S/P331S (IgG2m4) that were derived from hIgG4 [132]. When these mutations were introduced into a hIgG2 backbone it led to no detectable binding to hFcγRI, hFcγRIIIA or C1q; reduced binding to hFcγRIIB and no change in binding to FcγRIIA-H131 when compared to the WT hIgG2 antibody [132].

Another example of using hIgG4 based design to reduce effector functions are the V234A/G237A/P238S/H268A/V309L/A330S/P331S (IgG2c4d) mutations, where multiple residues within the hIgG2 constant region were replaced with IgG4 residues [134]. Introducing these IgG2c4d mutations resulted in no detectable binding to any FcγRs or C1q and no measurable ADCC, ADCP or CDC when compared to the WT hIgG2 counterpart [134].

Despite the choice of hIgG4 as an Fc-inert isotype, there is emerging evidence that the residual FcγR binding still has an effect. For example with anti-PD1 antibodies, residual FcγR binding has been implicated in the removal of the anti-PD1 mAb from the target cell by myeloid effectors [147]. This suggests that additional engineering may allow for further improvement of anti-PD1 mAb activity by reducing any remaining FcγR interactions. To this end, several additional mutations have been made in reagents that are approaching the clinic (see Section 5.4).

### 5.4. Anti-Cancer Antibodies with Reduced Effector Function Entering the Clinic

Perhaps the largest class of agents entering the clinic with reduced effector function are those targeting check-point molecules, with those targeting the PD1:PDL-1 axis the most prominent. Their role is to block the interaction between PD1 on effector T cells and PDL-1 on antigen presenting cells or tumor to allow the reactivation of tumor specific T cells [148]. As such, it is important that antibodies targeting PD1 do not result in the deletion of beneficial tumor reactive T cells. Consequently, all anti-PD1 mAb approved to date (camrelizumab, pembrolizumab, nivolumab, cemiplimab) as well as a number of others undergoing clinical evaluation (dostarlimab, spartalizumab and tislelizumab) are hIgG4 and include the S228P mutation to prevent F(ab) arm exchange [149].

Tislelizumab was further engineered as a hIgG4 anti-PD1 mAb with a number of additional mutations (E233P/F234V/L235A/D265A/R409K) to more fully abrogate FcγR interactions [150]. In a phase 2 trial in classical Hodgkin Lymphoma, tislelizumab achieved an ORR of 87%, compared to 71% for pembrolizumab in a separate trial in the same disease [151,152]. Whilst comparing results from separate trials should be done with caution, this hints that engineering anti-PD1 mAb to remove FcγR interactions may deliver additional benefit in the clinic. Tislelizumab, pembrolizumab and nivolumab have all been evaluated in squamous NSCLC. PFS for antibody with chemotherapy versus chemotherapy alone in their respective studies were: tislelizumab 7.6 versus 5.5 months, pembrolizumab 6.4 versus 4.8 months and nivolumab 7.1 versus 4.4 months [153,154,155], although the latter failed to meet its primary endpoint of improved overall survival (OS). As more data becomes available, the true benefit of Fc engineering for anti-PD1 antibodies should become clearer.

The other side of the PD1:PD-L1 axis has also been of interest with regards Fc-engineering, with three antibodies targeting PD-L1 currently approved: avelumab, atezolizumab and durvalumab. The latter two have been engineered to reduce FcγR interactions with the aim of enhancing blockade of PD1:PDL1 interactions without the deletion of APC or PD-L1 expressing T cells [156]. Atezolizumab is a hIgG1 containing the N297A mutation removing glycosylation to abrogate binding to all FcγR except FcγRI [40,157]. Durvalumab meanwhile contains a triple mutation (L234F/L235E/P331S) where binding is lost to all FcγR [131,158] Both atezolizumab and durvalumab resulted in modest increases in OS when combined with chemotherapy in separate SCLC trials compared to chemotherapy alone (12.3 versus 10.3 and 13.0 versus 10.3 months, respectively) [159,160]. As discussed above, evaluating the effect of the Fc modifications is difficult with no side-by-side comparisons having been performed. All three mAb have been approved for use in several malignancies, however it is perhaps noteworthy that both modified antibodies (atezolizumab and durvalumab) are approved for use in NSCLC whilst a trial with avelumab for the same indication did not show improvement in OS [156,161]. These antibodies are currently undergoing evaluation in a range of other tumors which may help to build a better picture as to the importance of Fc silencing when targeting PD-L1.

Outside of PD1:PDL-1, the use of Fc silencing has also been seen as an attractive strategy for the development of reagents targeting the emerging checkpoint molecule TIM-3. Two such reagents, Beigene BGB-A425 and Five Prime Therapeutics/BMS BMS-986258, are currently recruiting to early phase trials.

In addition to modulating effector functions, various groups have also attempted to understand and manipulate the half-life of antibodies [40,162,163,164,165,166,167,168,169,170,171,172].

## 6. Fc Engineering to Increase the Half-Life of Antibodies

**Figure 5:**
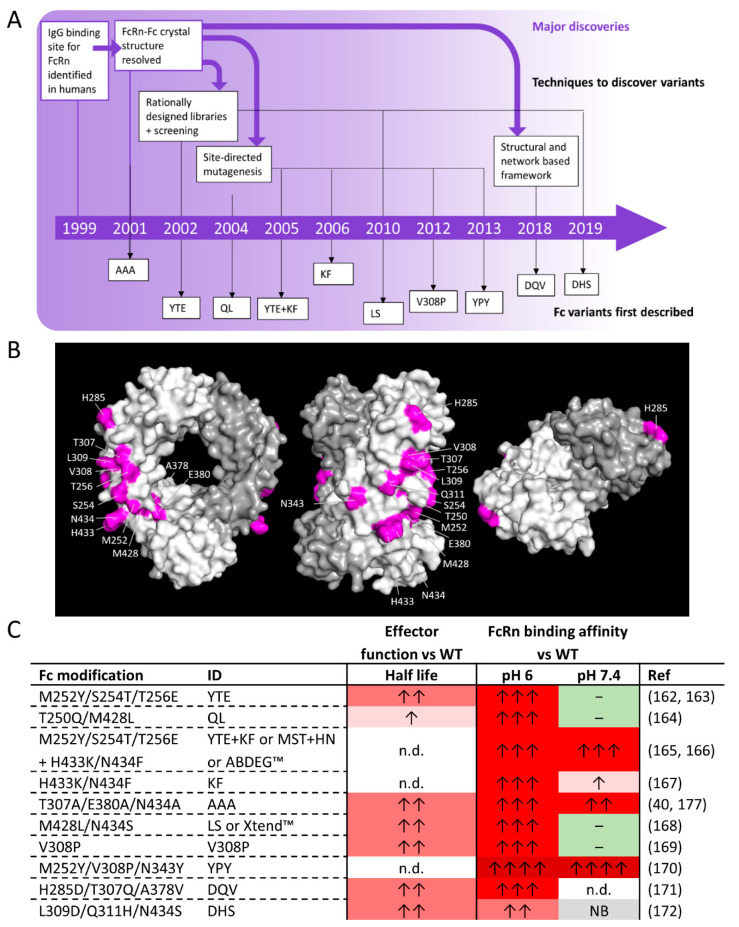
Fc modifications that improve half-life. (**A**) Timeline of events detailing when major discoveries were made regarding Fc:IgG interactions, techniques used to discover variants that have enhanced half-life and when the Fc variants were first described in the literature [40,162,163,164,165,166,167,168,169,170,171,172,177]. Letters refer to the common notation of the introduced amino acid substitutions as discussed in the main text. (**B**) Previously identified point mutations within the Fc that impact effector cell function annotated on a hIgG1 Fc crystal structure model (PDB: 3AVE; [49,50]) and detailed in the table below. (**C**) Magnitude of change to half-life or binding affinity for FcRn of variants were compared to WT antibodies of the same IgG isotype and F(ab) region. Each row depicts an independent experiment and therefore often independent methods, utilizing a different IgG backbone from a particular study as referenced in the last column. NB—no detectable binding, n.d.—no data, − no change, ↑ < 2 fold upregulation, ↑↑ 2–9.99 fold upregulation, ↑↑↑ 10–99.99 fold upregulation, ↑↑↑↑ > 100 fold upregulation.

Fc modification to modulate the binding affinity of IgGs to FcRn and thereby IgG half-life is of clear interest in various therapeutic and diagnostic scenarios. Perhaps of most relevance in cancer are Fc modifications that increase the efficiency with which IgG is recycled, extending half-life and allowing long term suppression of a therapeutic target whilst reducing the required dosing frequency. For example, extended treatment regimens using anti-CD20 mAb via maintenance strategies have helped to prolong remissions in certain lymphomas and so could benefit from longer half-lives [173,174]. Examples of these modifications have been summarized in Figure 5. However, it is clear that half-life extension may not be applicable for all anti-cancer antibodies, such as immunostimulatory mAb (e.g., TGN1412) where difficult to control adverse events such as cytokine storm responses may ensue [175]. Nonetheless, certain immune checkpoint inhibitor antibodies such as ipilimumab and avelumab have shown significant positive exposure-efficacy relationships with reduced clearance being associated with beneficial therapeutic responses [176]. However, it is important to note that for some immune checkpoint inhibitors, significant exposure-safety relationships were also reported, as demonstrated by increases in exposure correlating with increased risk of adverse events [176]. Therefore, risk:benefit relationships have to be carefully assessed for these targets, before half-life extension is considered. Perhaps for these reasons, to date, a limited number of clinically-relevant anti-cancer antibodies have adopted half-life extending technologies, as will be discussed in Section 6.1.

### 6.1. Amino Acid Substitutions Leading to Half-Life Extension.

The hIgG1 binding site for FcRn was mapped in humans in 1999 and 2 years later, the co-crystal structure was resolved alluding to the mechanism of pH-dependent binding via protonation of residues H310 and H435 [39,178]. Using this data, groups have engineered antibody variants that display higher affinity binding to FcRn and shown increased half-life (Figure 5). In order to extend mAb half-life, reagents need to have an increased affinity for FcRn at an acidic pH whilst still being efficiently dissociated at a neutral pH, a mechanism that presents a unique challenge for antibody engineering.

As already described, in 2001, analysis of the hIgG1 binding site for FcyRs and FcRn led to the design of many hIgG1 variants with different attributes, including enhanced binding to FcRn (Figure 5A) [40]. The triple mutation T307A/E380A/N434A displayed an 11.8-fold increase in FcRn binding at endosomal pH whilst retaining equivalent binding to the low affinity FcyRs when compared to WT hIgG1 [40]. In subsequent studies, it was found that, not only was binding to FcRn at endosomal pH enhanced, binding to FcRn at extracellular pH was also increased by 2.4-fold [177]. In vivo, the T307A/E380A/N434A mutant resulted in a 2.5-fold enhancement in half-life in a hFcRn transgenic mouse model compared to the WT hIgG1 antibody [177].

Dall’Acqua et al. used this knowledge to rationally design phage display libraries to identify other variants with increased binding affinity for FcRn (Figure 5A) [162]. They identified the triple mutation M252Y/S254T/T256E that enhanced FcRn binding by 11-fold at endosomal pH whilst not changing binding to FcRn at extracellular pH [162,163]. Subsequent crystal structure analysis of the mutations showed that the T256E mutation in hIgG1 results in the creation of 2 new salt bridges between the 256E residue of Fc-YTE and the Gln2 residue of the β2m subunit of FcRn that is thought responsible for enhanced binding to FcRn [179]. The Dall’Alcqua et al. cynomolgus monkey study demonstrated that the half-life of a M252Y/S254T/T256E IgG1 was 4-fold higher than WT antibody and when an initial dose of 30 mg/kg was given, the WT antibody was undetectable by day 55 whereas ∼60 μg/mL of M252Y/S254T/T256E IgG1 was detected in the serum at the same time-point [163]. However, introducing the M252Y/S254T/T256E mutations reduced binding to FcyRs and diminished their capacity for ADCC. Moreover, IgGs with M252Y/S254T/T256E mutations were found to have lower thermal stability by −8 °C when compared to WT hIgG1, which may affect antibody solubility and aggregation, both unfavorable properties for therapeutic antibodies [162,180].

Other half-life extending variants discovered through rational design and high-throughput screening include the M428L/N434S mutations [168]. In vitro, M428L/N434S hIgG1 was found to have an 11-fold enhancement in binding affinity to FcRn at pH 6 and no change in binding to FcRn at extracellular pH compared to WT hIgG1 [168]. Subsequent analysis demonstrated that the N434 residue in the Fc was involved in an unfavorable interaction with the L135 residue of FcRn and mutation of this residue (N434S) allowed the creation of additional hydrogen bonds with the carbonyls of FcRn [179]. In vivo, a M428L/N434S hIgG1 anti-VEGF antibody had a 3.2 fold longer half-life in hFcRn transgenic mice and a 3-fold longer half-life in cynomolgus monkeys [168].

Groups have also used site-directed mutagenesis for more targeted mutations based upon knowledge of the IgG binding site for FcRn (Figure 5A). Combined with analysis of molecular models of the hFc:FcRn complex crystal structures, Hinton et al. identified the T250Q/M428L amino acid substitutions that resulted in a 28-fold increase in binding to FcRn at endosomal pH whilst not affecting binding to FcRn at extracellular pH [164]. In vivo, a T250Q/M428L humanized IgG4 had a 1.9-fold increase in half-life and volume of serum of antibody cleared per unit of time was 2.8-fold lower in Rhesus monkeys compared to the WT humanized IgG4 [164]. Introducing the single mutation, V308P into hIgG1 also demonstrated a significant enhancement of binding (over 43-fold) to FcRn at endosomal pH, no change in binding to FcRn at extracellular pH and enhanced half-life in primates (2.4-fold increase in cynomolgus monkeys) compared to WT hIgG1 [169].

Other combinations of residues that were hypothesized to enhance binding to FcRn include two residues, H433 and N434, which are in close proximity to amino acids known to play a central role in human FcRn-IgG interactions [167]. Consequently, the double mutant H433K/N434F was assessed and found to have 16-fold enhanced binding to FcRn at endosomal pH while minimizing changes to FcRn binding at extracellular pH when compared to WT [167].

Using a structural model and a protein residue network to understand the interaction of Fc with FcRn at different pHs, Booth et al. sought variants specifically focusing on decreasing the k_off_ of the Fc:FcRn interaction at pH 6 while maintaining binding to FcyRs [171]. Through their framework they identified a triple mutant H285D/T307Q/A378V that enhanced binding to FcRn at endosomal pH by over 13-fold and enhanced half-life by over 8-fold in hFcRn transgenic mice, whilst enhancing binding to all FcyRs and C1q and enhancing relevant Fc effector functions (ADCC and CDC) compared to WT [171].

Another mechanism to enhance half-life of antibodies has been to design antibody variants with enhanced binding of FcRn at endosomal pH while abrogating binding at extracellular pH. Residual binding of IgG at pH 7.4 has been shown to negatively affect plasma recycling and circulation persistence [172,181]. This process would be beneficial for antibodies that require release into the plasma and persistence there, for example, antibodies targeting cell-bound targets and where removal of any residual binding of IgGs at extracellular pH may be favorable [97]. Lee et al. rationally designed libraries for high-throughput screening in escherichia coli in order to identify hIgG1 Fc mutations that bind selectively to FcRn at pH 5.8 but not at pH 7.4 [172]. Through these screening strategies they found the triple mutant L309D/Q311H/N434S had a 5-fold enhancement in binding to FcRn at endosomal pH and no binding to FcRn at extracellular pH compared to WT hIgG1 [172]. In vivo, half-life of L309D/Q311H/N434S hIgG1 was enhanced by over 4-fold in hFcRn/hβ2m/hFcγR/hIgG1,κ mice compare to WT hIgG1 [172]. Unlike the M252Y/S254T/T256E triple mutation, antibodies with L309D/Q311H/N434S mutations retained FcyR binding and ADCC capacity [172].

The aforementioned Fc variants were discovered by studies focused on enhancing binding to FcRn at endosomal pH without changing or minimizing changes to binding at extracellular pH, however, variants can also be designed to modulate binding at extracellular pH. One rationale for identifying antibody variants with differential binding to FcRn at extracellular pH is to generate so-called “sweeping antibodies” to clear away soluble targets. An undesirable repercussion of targeting soluble antigens using antibodies is that it can paradoxically lead to a >1000-fold increase in the antigen [182,183]. This is due to the half-life of IgG being much longer than that of the antigen being bound, allowing an accumulation of antibody-antigen complexes and thus an increase in plasma antigen presence due to the reduction in clearance [170]. To counteract this, sweeping antibodies are engineered to enhance binding to FcRn at both endosomal and extracellular pH. Increasing binding to FcRn at pH 7.4 stops the antibody being released into the plasma, allowing it to persist in complex with the soluble antigen [170]. Instead, antibodies are tightly bound to the endosomal membrane and bind to ligands at the cell surface [170]. As these antibodies still remain bound to the cell surface the antibody:antigen complex can be quickly internalized into the cell. Sweeping antibodies are often engineered to be able to dissociate from the antigen once internalized in the endosome thus targeting the antigen alone for lysosomal degradation [184]. As the antibody is kept at the cell surface due to an enhanced binding to FcRn at extracellular pH and kept in the endosome due to strong binding to FcRn at endosomal pH, the sweeping antibody is not released into the plasma to form antibody-antigen complexes or degraded in the lysosome and is instead constantly recycled within the cell [184]. Therefore, these antibodies are highly effective at clearing soluble targets coupled to an extended half-life. With regards anti-cancer antibodies, these sweeping antibodies could be valuable in targeting immunosuppressive cytokines such as TGF-β [185]. For example, antibodies against these cytokines paired with immune checkpoint inhibitors such as anti-PD-1/PD-L1 antibodies have been shown to enhance anti-tumor activity [185]. Sweeping antibodies could more effectively clear these soluble targets and thereby reverse immune suppression. Although sweeping antibodies have not yet been adopted for cancer treatment, Fc modifications to produce these reagents are described below and could be implemented in the future.

Using molecular modelling, residues in the hIgG1 Fc, near to the FcRn binding site, were identified as candidates to mutate in order to alter binding to FcRn. The resulting mutations M252Y/S254T/T256E + H433K/N434F (referred to as YTE-KF or MST-HN or Abdeg) were found to have a 20-fold increase in binding to FcRn at both endosomal and extracellular pH [165].

Another example of mutations incorporated into hIgG1 that are able to enhance binding to FcRn at both an endosomal and extracellular pH include the M252Y/V308P/N343Y mutations. The M252Y/V308P/N343Y mutations were discovered by Igawa et al. and were found to enhance binding to FcRn at endosomal pH by >400-fold and extracellular pH by >2500-fold compared to WT hIgG1. In vivo, hIgG1 anti-IL-6R antibodies with the M252Y/V308P/N343Y mutations reduced total human soluble IL-6R in the plasma by 1000-fold when compared to the WT hIgG1 antibody in hFcRn transgenic mice [170].

### 6.2. Anti-Cancer Antibodies with Half-Life Extension Entering the Clinic

As described above, Fc-engineering to improve half-life will endow mAb with prolonged bioavailability and potentially therapeutic effect, alongside reduced dosing frequency requirements. In pre-clinical studies cetuximab and bevacizumab engineered with Xencor’s Xtend technology had a substantially improved half-life compared to the unmodified mAb which translated into more prolonged tumor control [168]. To date, only one extended half-life antibody has been approved by the FDA. Ravulizumab is an engineered version of eculizumab, targeting C5, for use in paroxysmal nocturnal hemoglobinuria and atypical hemolytic uremic syndrome [186]. The introduced amino acid substitutions (VH_Y27H, VH_S57H, M428L, N434S) resulted in a 4-fold extension in half-life allowing dosing to be reduced from every 2 weeks to every 8 weeks whilst maintaining efficacy [187]. To date Fc enhancing technology for anti-cancer reagents has been limited to a panel of checkpoint blockade targeting bispecific mAb generated by Xencor using Xtend technology (Xmab23104: PD-1 x LAG3, XmAb22841: CTLA-4 x LAG3 and XmAb20717: PD-1 x CTLA-4) and no whole IgG molecules have progressed. This limited interest in extended half-life antibodies for cancer is likely due to the natural half-life (~21 days) being sufficient in most contexts, for example when using mAb as part of a chemoimmunotherapy regimen. However, there may be occasions where enhanced exposure may be of particular benefit, such as within a maintenance regime.

## 7. Conclusions

To date, the most clinically advanced Fc engineered mAb are those designed to have increased effector function, predominantly through enhanced engagement with activatory FcγR. The most advanced molecules in the clinic have relied on glycomodification, through the removal of core fucose residues to enhance effector cell function. Whilst this has not resulted in a large step change in therapeutic outcome, the success of reagents such as obinutuzumab, mogamulizumab and margetuximab has delivered important proof of concept that Fc-engineered mAb can elicit patient benefit. Other mAb under development will likely build on this success, particularly with the development of antibodies based around the S239D/I332E mutations, found to offer the most substantial enhancement of ADCP and ADCC. The detrimental role of FcγR engagement to the activity of mAb targeting the checkpoint inhibitory receptor PD-1 appears clear and appropriately engineered Fc-silenced reagents have progressed to approval. In general this has relied upon the use of the IgG4 isotype mutated to prevent F(ab) arm exchange with all IgG4 mAb in the clinic incorporating the S228P mutation. It will be important to understand if introducing additional Fc-silencing mutations into IgG4 mAb will offer robust clinical benefit relative to introducing silencing mutations into the more widely used IgG1 isotype. One of the remaining challenges with antibody engineering is to understand the balance between increasing antibody activity and unwanted toxicity; a problem that may be most acute for immunostimulatory antibodies or those with an extended half-life. With an ever-increasing range of Fc engineered variants entering the clinic, fully evaluating the relative merits of these changes will be of increasing importance in order to deliver the greatest patient benefit.

## Figures and Tables

**Table 1:** Mutations augmenting cellular effector function and FcγR binding.

		Effector Function vs. WT		Binding Affinity vs. WT					
Fc Modification	ID	ADCC	ADCP	FcγRIIA^H^	FcγRIIA^R^	FcγRIIB	FcγRIIIA^F^	FcγRIIIA^V^	Ref
S298A/E333A/K334A	AAA	↑	n.d.	↓↓	n.d.	↓↓	↑↑	↑	[40]
Afucosylation (Potelligent)	Potelligent	↑↑	n.d.	n.d.	n.d.	n.d.	↑↑↑	n.d.	[41]
S239D/I332E	DE	↑↑	↑	n.d.	n.d.	↑↑↑	↑↑↑	↑↑↑↑	[42]
S239D/A330L/I332E	DLE	↑↑↑	↑	n.d.	n.d.	↑↑↑	↑↑↑	↑↑↑↑	[42]
G236A	G236A	−	↑	↑↑	↑↑	↑	−	−	[43]
G236A/S239D/I332E	ADE	↑	↑	↑↑	↑↑↑	↑↑↑	↑↑↑	↑↑↑	[43]
G236A/A330L/I332E	GAALIE	d.n.s.	n.d.	↑↑	↑↑	↓↓	↑↑	↑↑	[44]
G236A/S239D/A330L/I332E	GASDALIE	n.d.	n.d.	↑↑↑	↑↑↑	↑	↑↑↑	↑	[45]
F243L/R292P/Y300L/V305I/P396L	LPLIL	↑↑	n.d.	↑↑	↑	↑	↑↑↑	↑↑↑	[46]
L235V/F243L/R292P/Y300L/P396L	VLPLL	↑	n.d.	↑	↓↓	↓↓	↑↑	↑	[47]
One heavy chain: L234Y/L235Q/G236W/S239M/H268D/D270E/S298A & opposing heavy chain: D270E/K326D/A330M/K334E	Asym-mAb1	↑↑	n.d.	↑↑	↑↑	−	↑↑↑↑	↑↑↑↑	[48]

Magnitude of change in effector function (fold change) or binding affinity of variants were compared to WT antibodies of the same IgG isotype and F(ab) region. Each row depicts an independent experiment and often independent methods, utilizing a different IgG backbone from a particular study as referenced in the last column. NB = no detectable binding, n.d. = no data, d.n.s. = data not shown, − = no change, ↑ <2 fold increase, ↑↑ 2–9.99 fold increase, ↑↑↑ 10–99.99 fold increase, ↑↑↑↑ >100 fold increase, ↓↓ 2–9.99 fold decrease. **FcγRIIA^H^** FcγRIIA-H131, **FcγRIIA^R^** FcγRIIA-R131, **FcγRIIIA^F^** FcγRIIIA-F158, **FcγRIIIA^V^** FcγRIIIA-V158.

**Table 2:** Fc engineered antibodies currently approved for clinical use or in late stage development.

Antibody	Manufacturer	Target	Fc Modification	Modification Effect	Indication(s)	Stage
Obinutuzumab	Roche	CD20	Afucosylation	Increased ADCC	B cell lymphoma	Approved: USA—2013 EU—2014
Mogamulizumab	Kyowa Hakko Kirin	CCR4	Afucosylation	Increased ADCC	T cell lymphoma	Approved:
						USA and EU—2018
Margetuximab	MacroGenics	HER2	L235V/F243L/R292P/Y300L/P396L	Increased ADCC	Breast cancer	Phase 3
Ublituximab	LFB/TG Therapeutics	CD20	Afucosylation	Increased ADCC	NHL	Phase 3
Tafasitamab	MorphoSys	CD19	S239D/I332E	Increased ADCC	Hodgkin lymphoma	Approved: USA—2020
						Under review in EU
Zalifrelimab	Agenus	CTLA-4	S239D/A330L/I332E	Increased ADCC	Advanced solid tumours	Phase 2
BI 836826	Boehringer Ingelheim	CD37	S239D/I332E	Increased ADCC	DLBCL	Phase 2
Ocaratuzumab	Mentrik Biotech	CD20	P247I/A339Q	Increased ADCC	Leukaemia/lymphoma	Phase 2 *
Durvalumab	AstraZeneca	PD-L1	L234F/L235E/P331S	Fc disabled	Urothelial carcinoma	Approved: USA—2017
					NSCLC	EU—2018
Atezolizumab	Roche	PD-L1	N298A	Fc disabled	Urothelial carcinoma	Approved: USA—2016 EU—2017
					NSCLC	
					SCLC	
					Breast cancer	
APL502	Apollomics	PD-L1	Unknown	Fc disabled	Squamous cell carcinoma H&N	Phase 3

NB: F(ab) arm exchange modified IgG4 antibodies have not been included. Antibodies engineered for increased ADCC are highlighted in red and those with Fc disabling modifications are in blue. Regulatory status obtained from The Antibody Society [68] (and is correct as of 13/08/2020. * Phase 2 trial of ocaratuzumab completed in 2011 with no further information available.

**Table 3:** Reduction in effector function or binding affinity to FcγRs in Fc-silenced variants compared to WT antibodies.

		Effector Function vs WT			Binding Affinity vs WT							
Fc Modification	ID	ADCC	ADCP	CDC	FcγRI	FcγRIIA^H^	FcγRIIA^R^	FcγRIIB	FcγRIIIA^F^	FcγRIIIA^V^	C1q	Ref
Aglycosylation (N297A/Q/G)	NA	↓	n.d.	↓↓	↓↓↓	NB	NB	NB	NB	NB	NB	[57,124,125,126]
L235A/G237A/E318A	AAA	↓↓	n.d.	n.d.	↓↓**						n.d.	[127]
L234A/L235A	LALA	↓↓	n.d.	↓↓	↓↓↓↓	NB *		NB	NB *		NB	[128,129]
S228P/L235E	IgG4-PE	None	n.d.	n.d.	↓↓↓↓	↓↓↓	↓↓↓	↓↓	n.d.	NB	NB	[130]
G236R/L328R	RR	n.d.	n.d.	n.d.	↓↓↓↓	NB	n.d.	NB	n.d.	NB	n.d.	[115]
S298G/T299A	GA	n.d.	n.d.	n.d.	↓↓↓	↓	↑↑	↑	NB	NB	NB	[124]
L234F/L235E/P331S	FES	n.d.	n.d.	n.d.	NB	↓↓*		n.d.	n.d.	↓↓↓	↓↓↓	[131]
H268Q/V309L/A330S/P331S	IgG2m4	n.d.	n.d.	n.d.	NB	−	n.d.	↓↓	NB	NB	NB	[132]
E233P/L234V/L235A/G236del/S267K	XmAb^®^ bispecific	n.d.	n.d.	n.d.	NB	NB	NB	NB	n.d.	NB	n.d.	[133]
L234A/L235A/P329G	LALA-PG	None	n.d.	n.d.	NB	NB	NB	↓↓↓↓	n.d.	NB	NB	[130]
V234A/G237A/P238S/H268A/V309L/ A330S/P331S	IgG2c4d	None	None	None	NB	NB *		NB	NB *		NB	[134]
L234F/L235E/D265A	FEA	n.d.	n.d.	n.d.	NB	NB **			NB *		NB	[135]

Magnitude of effector function (fold-change) or binding affinity of variants were compared to WT antibodies of the same IgG isotype and F(ab) region. Each row depicts an independent experiment and therefore often independent methods, utilizing a different IgG backbone from a particular study as referenced in the last column.* allotype not specified in methods. ** specific binding affinity for each FcγR member not analyzed. NB = no detectable binding, n.d. = no data, − = no change, ↑ < 2 fold upregulation, ↑↑ 2–9.99 fold upregulation, ↓ <2 fold downregulation, ↓↓ 2–9.99 fold downregulation, ↓↓↓ 10–99.99 fold downregulation, ↓↓↓↓ > 100 fold downregulation, **FcγRIIA^H^** FcγRIIA-H131, **FcγRIIA^R^** FcγRIIA-R131, **FcγRIIIA^F^** FcγRIIIA-F158, **FcγRIIIA^V^** FcγRIIIA-V158.
